# Artificial intelligence-driven virtual rehabilitation for people living in the community: A scoping review

**DOI:** 10.1038/s41746-024-00998-w

**Published:** 2024-02-03

**Authors:** Ali Abedi, Tracey J. F. Colella, Maureen Pakosh, Shehroz S. Khan

**Affiliations:** 1 KITE Research Institute, Toronto Rehabilitation Institute, University Health Network, Toronto, ON Canada; 2 Library & Information Services, Toronto Rehabilitation Institute, University Health Network, Toronto, ON Canada; 3 Institute of Biomedical Engineering, University of Toronto, Toronto, ON Canada

**Keywords:** Rehabilitation, Computer science

## Abstract

Virtual Rehabilitation (VRehab) is a promising approach to improving the physical and mental functioning of patients living in the community. The use of VRehab technology results in the generation of multi-modal datasets collected through various devices. This presents opportunities for the development of Artificial Intelligence (AI) techniques in VRehab, namely the measurement, detection, and prediction of various patients’ health outcomes. The objective of this scoping review was to explore the applications and effectiveness of incorporating AI into home-based VRehab programs. PubMed/MEDLINE, Embase, IEEE Xplore, Web of Science databases, and Google Scholar were searched from inception until June 2023 for studies that applied AI for the delivery of VRehab programs to the homes of adult patients. After screening 2172 unique titles and abstracts and 51 full-text studies, 13 studies were included in the review. A variety of AI algorithms were applied to analyze data collected from various sensors and make inferences about patients’ health outcomes, most involving evaluating patients’ exercise quality and providing feedback to patients. The AI algorithms used in the studies were mostly fuzzy rule-based methods, template matching, and deep neural networks. Despite the growing body of literature on the use of AI in VRehab, very few studies have examined its use in patients’ homes. Current research suggests that integrating AI with home-based VRehab can lead to improved rehabilitation outcomes for patients. However, further research is required to fully assess the effectiveness of various forms of AI-driven home-based VRehab, taking into account its unique challenges and using standardized metrics.

## Introduction

Rehabilitation aims at providing interventions to patients to improve recovery, reduce disability, and optimize functioning and health outcomes^1^. Rehabilitation generally involves prescribed exercises, education, and counseling sessions, as well as in-person interactions with a clinician. There can be several impediments to traditional in-person rehabilitation, including transportation needs, appointment scheduling conflicts^2^, financial constraints^3^, and staff shortages in the healthcare sector^4,5^. Up to 50% of women tend to drop out of their rehabilitation program in many patient populations, due to these issues and other social and cultural factors^6^. During the COVID-19 pandemic, most rehabilitation centers either ceased to operate or worked at a limited capacity, thus severely impacting millions of patients worldwide^7^. As a result, traditional in-person rehabilitation is being stretched to its limits, and many people (especially older adults) may not be able to access these services to improve their physical and mental well-being.

With the increasing adoption of internet services in major urban areas, virtual rehabilitation (VRehab) or synonymously Telerehabilitation is becoming more prevalent and mainstream^8–11^. Previous research has demonstrated that home-based VRehab provides similar health outcomes to in-person rehabilitation and is better than no rehabilitation^12–17^. VRehab focuses on improving patients’ physical and mental health and quality of life through home-based virtual exercise and therapy sessions. During VRehab sessions, clinicians and researchers often utilize technologies that generate complex and large single- or multi-modal datasets, which require new analysis methods to support patients’ recovery. The use of technology creates opportunities for Artificial Intelligence (AI) to be utilized in the VRehab setting^8,18–20^ to address research questions involving assessment^21^, recognition^22,23^, and prediction^24,25^ of various patient health outcomes. Applications of AI in VRehab include but are not limited to patient’s movement and physical activity analysis, physical exercise assessment^21^, pain detection and measurement^26,27^, affective state analysis^23^, and compliance prediction^24^.

### Why home-based VRehab?

In traditional rehabilitation programs involving in-person hospital/clinic visits, the presence of clinicians is required at different stages of the program, necessitating that patients commute to and from the hospital or clinicians travel to patients’ homes or long-term care homes. This imposes several barriers to the successful completion of the program among patients,^2,3,5,7,28,29^ including: (i) Transportation constraints pose difficulties for patients with disabilities and older adults; (ii) Patients residing in remote regions may lack access to nearby rehabilitation centers, requiring them to undertake long-distance travel to participate in rehabilitation programs; (iii) The rehabilitation sector experiences a shortage of staff, leading to scheduling limitations and conflicts resulting in further delays in recovery; (iv) In-person participation becomes particularly challenging during pandemic situations that enforce social distancing measures. Consequently, patient enrolment rates may be lower and dropout rates may be high; thus preventing patients from successfully integrating into their community and living independently^6,30–32^.

On the other hand, VRehab aims to deliver rehabilitation programs virtually to patients’ homes and has the potential to overcome many barriers to program attendance and completion^28,29^. Integrating AI into VRehab to automate different stages of rehabilitation holds significant potential for complementing clinicians and improving the quality of care they provide to patients in their homes. AI-driven VRehab platforms offer promising solutions for addressing the shortage of rehabilitation staff and optimizing operational efficiencies. By delivering rehabilitation services virtually to patients’ homes, VRehab expands access to healthcare for diverse populations, including those who are underrepresented and reside in remote communities without access to rehabilitation centers^2,3,5,7^. However, for VRehab to be effective, patients need access to computers or smart devices, sensors, and an internet connection at home. Additionally, patients should be digitally literate and familiar with technological infrastructures. A detailed discussion of these limitations can be found in the discussion section.

### What role can AI play in VRehab?

**Fig. 1:**
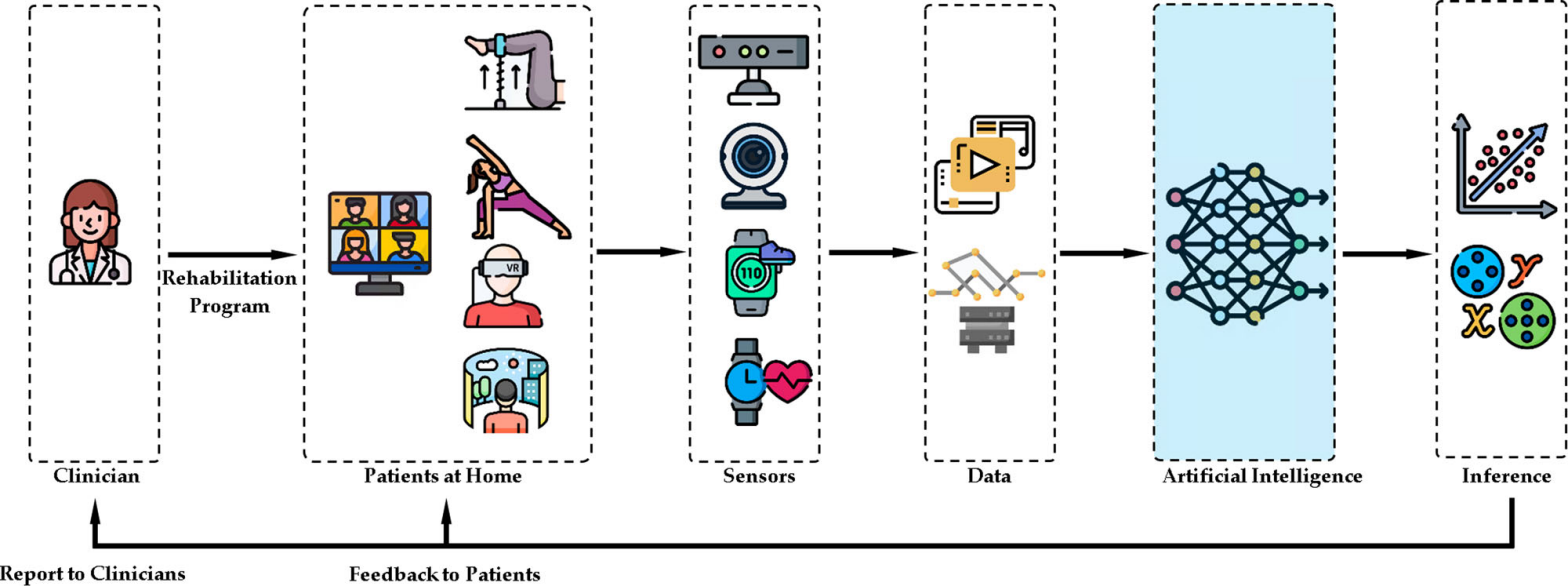
A conceptual diagram depicting various stages of AI-driven VRehab platforms. This scoping review focuses on AI algorithms, which is highlighted in blue.

Figure 1 illustrates various stages of a general AI-driven VRehab program. VRehab programs typically include a clinical assessment and clinician meetings with patients virtually or in person, and then the prescription of individualized VRehab programs. Usually, these programs include regular educational sessions^8–10^ and aerobic and resistance training exercises^21^ targeting improvement of function and mobility as well as avoiding sedentary lifestyles^33,34^. A variety of sensing devices may be used to conduct the initial clinical assessment virtually at home, and subsequently collect physiological, ambient, and contextual data from patients at home during VRehab sessions^8^. For instance, a webcam/camera on a personal computer or smartphone can be used to capture videos of patients while performing rehabilitation exercises which could provide important information on their functional recovery. A smartwatch with a built-in accelerometer can provide vital data on mobility parameters, including the number of steps taken and sedentary lifestyle^35,36^. These single or multi-modal data can be used to build AI algorithms for measuring patients’ overall improvements in their rehabilitation program and providing feedback, resources, and encouraging notifications to patients to complete their programs successfully.

AI algorithms using sensor data to make inferences about various patient health outcomes can be classified into three main approaches: end-to-end, feature-based, and hybrid^10^. End-to-end approaches involve employing deep learning-based artificial neural networks to make inferences using raw sensor data. On the other hand, feature-based approaches involve extracting features from raw sensor data, which are then utilized by machine learning or deep learning models to make inferences. In feature-based approaches, clinical domain knowledge may be utilized to extract or select the most suitable features for specific inference tasks^21^. Hybrid approaches combine the two approaches described above. As an example, raw video data of patients^19^ during VRehab sessions can be analyzed by deep-learning models (in an end-to-end approach) or the eye gaze direction, head movements, and range of motions as features extracted from the raw video data^37^ can be analyzed by machine-learning or deep-learning models (in a feature-based approach) to make inferences about patients’ emotions and behaviors^38^.

Prior to deploying AI algorithms on VRehab platforms for making inferences about patients’ health outcomes, it is essential to train them using relevant data. To illustrate, in assessing exercise quality, annotated data of previous patients performing both correct and incorrect exercises^21^ can be used to train the AI algorithms^10,39^. Once the algorithms are trained, they can be deployed on VRehab platforms to automatically assess exercise quality for new patients^10,38,39^. Inferences made by trained AI algorithms can be utilized in a variety of ways^8–10^. For example, the results of the measurement of the correctness of exercises can be input to a virtual coach (avatar) on a computer screen to provide real-time feedback and guidance for patients to correct their technique and movements in order to complete the exercises correctly^40–42^. The number of steps taken each day can be reported to the patient/clinician through the VRehab platform^43^. In the case of a low step count and a sedentary lifestyle, the patient would then receive customized notifications on the VRehab platform and/or specific instructions from the VRehab clinician.

### Related reviews

Several recent reviews have been conducted on the applications of AI in the rehabilitation of different populations^8,10,18,19,44–46^ as well as VRehab or telerehabilitation^9,47–50^. None of the published literature has addressed the combined role of AI and VRehab to support patient recovery in various rehabilitation populations. AI in VRehab is an emerging field; this scoping review is timely to understand and analyze the existing results, challenges, and future directions to help improve the health outcomes of rehabilitation patients living in the community.

AI plays a primary role in VRehab by analyzing patient data collected by various sensing devices at patients’ homes remotely and making inferences regarding their recovery and health outcomes. It facilitates the automation of rehabilitation programs and permits the delivery of these programs to patients in their homes. In this paper, a scoping review was conducted to methodologically map the current research on the applications of AI in VRehab, and to identify existing gaps in knowledge and associated challenges. In order to gain a comprehensive understanding of the field, all adult (>age 18) patient populations and different types of rehabilitation were considered. The following research questions guided this scoping review: (1) How was AI applied in the delivery of home-based VRehab programs to patients living in the community? (2) How effective was the application of AI in the delivery of home-based VRehab programs for patients living in the community?

## Methods

### Design

This study used a scoping review methodology due to the broad nature of the research questions, the heterogeneity of the studies and the populations, as well as the lack of comprehensive reviews conducted previously^51,52^. The scoping review was conducted using the framework proposed by Arksey and O’Malley^51^ and reported in accordance with the Preferred Reporting Items for Systematic Reviews and Meta-Analyses extension for Scoping Reviews (PRISMA-ScR) Checklist^52^.

### Eligibility criteria

#### Inclusion criteria

Peer-reviewed journal and conference articles written in English which conducted quantitative, qualitative, and mixed-method studies were included. For inclusion in the review, studies had to present the development of a new research or commercial AI-driven platform or use a previously developed AI-driven platform for the delivery of rehabilitation services to patients at home. In order to be considered for inclusion in the review, the platform must meet all three criteria listed below: (i) The platform had to be AI-driven, i.e., machine-learning and or deep-learning algorithms had to be incorporated into the platform for the purposes of making inferences about patient health outcomes. (ii) The platform had to be evaluated on adult patients aged 18 or older undergoing any type of rehabilitation program. (iii) The platform had to be evaluated on patients in their homes in a fully home-based or hybrid (home- and hospital-based) rehabilitation program. Therefore, in the SPICE framework^53^, setting, population, intervention, comparison, and evaluation were patients’ homes, adult patients, any rehabilitation program, technology/AI algorithms, and effectiveness, respectively.

#### Exclusion criteria

Non-peer-reviewed and non-English publications or resources were excluded. Studies were excluded if they (i) did not incorporate AI into their rehabilitation platform, (ii) did not evaluate their platform on patients, or (iii) did not evaluate their platform in patients’ homes. If one or more of the above criteria were met, studies were excluded. It is to be noted that some AI and VRehab solutions may be delivered in a hospital or clinic setting. While these are useful to many patients, these approaches may still suffer from the barriers of in-person attendance and constant clinical supervision (as discussed in the introduction section). A large number of the studies reviewed developed AI-driven VRehab platforms, however, they only tested them on healthy participants, clinicians, students, or research team members. Those studies were deemed out of scope for our review as we emphasize on improving health outcomes for patients living in the community using AI-driven VRehab solutions. Studies in which video games, virtual reality, or augmented reality were used to deliver VRehab without the application of AI methods were also excluded.

### Information sources and search strategy

In order to identify relevant studies, a comprehensive literature search was developed in collaboration with a Library Sciences Expert (M.P.) and subsequently refined through team discussion. A.A. and T.J.F.C. provided M.P. with an initial list of keywords along with a list of 25 representative relevant papers that must be retrieved from databases. Subsequently, M.P., A.A., and T.J.F.C. refined the keyword list and formulated a search strategy for individual databases. An extensive search was conducted in several bibliographic electronic databases, including PubMed/MEDLINE, Embase, IEEE Xplore, and Web of Science, from inception to June 2022. Furthermore, a grey literature search was conducted on Google Scholar in order to identify and include studies published between June 2022 and June 2023. Box 1 presents the unique search keywords used to search the databases. The exact search strategy and the keywords associated with the search in all the databases are available in Supplementary Table 1. The search results were exported as multiple XML files, merged, imported into the Covidence web application for systematic review^54^, and duplicates were removed. The reference lists of included studies were searched to identify any additional relevant studies.

Box 1Unique keywords used to search the databases rehabilitation, cardiac rehabilitation, stroke rehabilitation, occupational therapy, physical therapy, exercise therapy, telerehabilitation, rehab, tele-rehab, virtual rehab, e-rehab, therapy, physiotherapy, kinesiotherapy, remote consultation, home care services, home, virtual, in-home, at-home, web-based, internet, tele-consult, teleconsult, remote, environment, monitoring, artificial intelligence, machine learning, algorithms, pattern recognition, automated, signal processing, computer-assisted, affective, computational, ambient intelligence, deep learning, algorithm, sensing system, wearable, physiology sensor, computer vision, artificial neural network, motion data, recognition, locomotive, gesture, automatic, pain, engagement, pattern, active, technology, sensor, device, monitor, Kinect, video, camera, action, technology solution, physical action, feedback, data motion stride, motion capture, tracking.

### Selection of sources of evidence

A group of three independent reviewers, namely A.P., H.P., and Z.K., was involved in conducting the title and abstract screening using the Covidence web application. Each study underwent review by at least two of these reviewers. Subsequently, the relevant studies were subjected to a full-text review and data charting, which were carried out by at least two reviewers chosen from a group including A.A., A.P., H.P., and Z.K. Any conflicts that arose during the title and abstract screening phase were resolved by at least one independent reviewer, chosen from A.A. and S.S.K., and during the full-text review phase were resolved by S.S.K.

### Data charting process and data items

In order to address the research questions for this scoping review, a data charting form was developed to extract relevant information from the screened studies. The data charting form comprised of four sections: (i) study characteristics, participants, and settings, (ii) study aims, methodologies, and key findings, (iii) characteristics of VRehab programs, and (iv) AI algorithms and their applications.

### Synthesis of results

To address the research questions, a descriptive analysis was conducted followed by a summary of relevant study characteristics in narrative form using tables. Studies were sorted by year and analyzed based on the data characteristics described above. Due to heterogeneity in patient populations, rehabilitation types, outcome measurement tools, and measurement times, a meta-analysis was not conducted^55^.

## Results

### Selection of sources of evidence

**Fig. 2:**
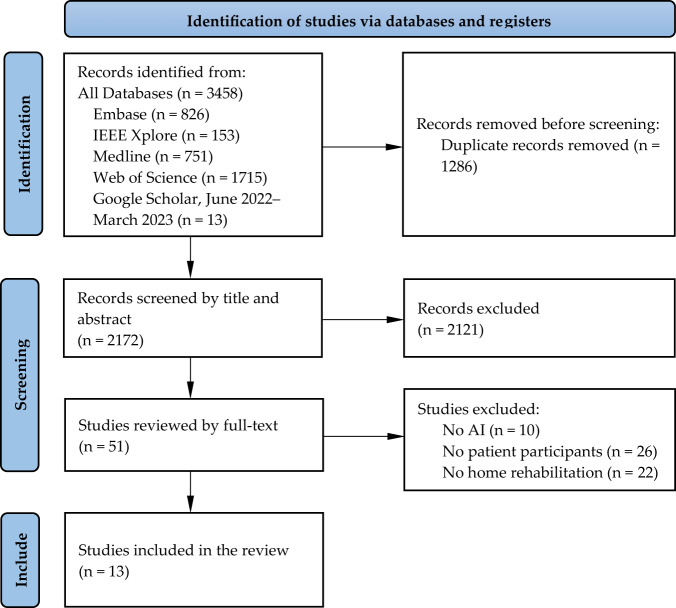
PRISMA flow diagram for the scoping review. Of the 2172 unique titles and abstracts initially screened, 51 full-text studies were further evaluated, resulting in 13 studies being included in the scoping review.

Figure 2 illustrates the PRISMA flow diagram, which describes the study selection process. Upon removing duplicates, a total of 2172 studies were identified through comprehensive literature searches of electronic databases and grey literature. Following title and abstract screening, 2121 studies were excluded, and 51 full-text studies were retrieved for full-text review. Among these 51 studies, 38 were excluded due to the absence of any one or more of the following three inclusion criteria: (i) using AI in the VRehab platform; (ii) evaluating the platform within a patient population; and (iii) evaluating the platform at home. This resulted in the inclusion of 13 studies.

### Characteristics of sources of evidence

**Fig. 3:**
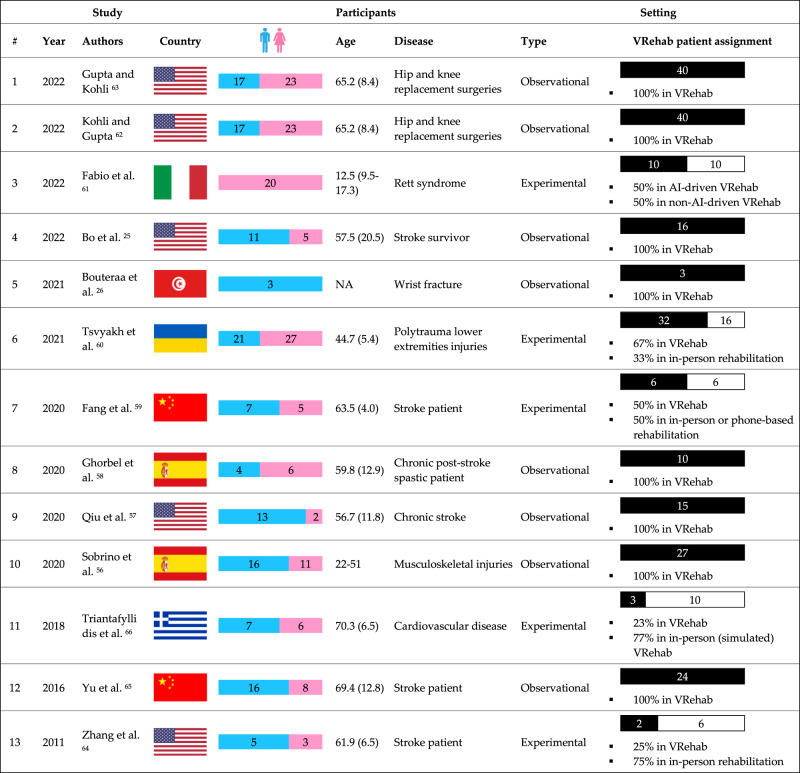
Study characteristics, participants, and settings in the included studies. The figure outlines the characteristics of the studies included in this scoping review.

Figure 3 and Tables 1–3 outline the characteristics of the studies included in this scoping review. An “NA" in the tables indicates that the corresponding item was not addressed or discussed in the paper.

**Table 1:** The aim, methodology, and key findings of the included studies.

Authors	Aim	Methodology	Key findings
Gupta and Kohli^63^	To evaluate the effectiveness of VRehab on hospital readmission rate.	Using AI and computer vision, the TheraNow smartphone application provided exercise-based rehabilitation plans for patients and assessed the quality of their exercises.	Using AI-driven VRehab resulted in lower hospital readmission rates.
Kohli and Gupta^62^	To evaluate patients' level of satisfaction and likelihood of recommending the VRehab platform to others.	Using AI and computer vision, the TheraNow smartphone application provided exercise-based rehabilitation plans for patients and assessed the quality of their exercises.	Patients reported high levels of satisfaction with the VRehab platform.
Fabio et al.^61^	To compare the performance of patients in non-AI-driven VRehab and AI-driven VRehab.	While non-AI-driven VRehab was simple video communication between patients and clinicians, AI-driven VRehab was equipped with eye gaze and body skeleton acquisition. The eye gaze and skeleton data were observed by the clinician to understand patients' interaction, attention, and movements.	AI-driven VRehab resulted in improvements in a few neuropsychological measurements.
Bo et al.^25^	To establish a progressive framework for predicting rehabilitation outcomes.	Patients' motion data was collected using the built-in sensors in their smartphones. AI algorithms were used to analyze patients' motion data and demographic information to predict rehabilitation outcomes.	Combining clinical and demographic data with movement data significantly improved the performance of predictive AI algorithms.
Bouteraa et al.^26^	To develop predictive models to estimate pain using features extracted from various sensors and use the estimated pain in the control loop for generating safe robot actions.	By using a computer vision system, the physiotherapist’s gestures were translated into commands for the robot. As a measure of safety, if the pain level exceeded a certain threshold, the robot would stop the action, even if the desired angle had not yet been reached.	The developed human-robot interface was able to provide a control and monitoring interface for home-based VRehab.
Tsvyakh et al.^60^	To implement an AI-driven VRehab platform and compare it with traditional rehabilitation.	Different sensors were used to collect data from patients, including exercise time, local temperature, and the biomechanics of active movements of the injured limb. The collected data was accessible to clinician surgeons to monitor patients.	Compared to traditional rehabilitation, VRehab reduced the time that surgeons spent consulting with their patients and resulted in higher levels of patient satisfaction.
Fang et al.^59^	To longitudinally examine the efficacy of VRehab.	Wearable sensors collected accelerometer data from patients while they performed rehabilitation exercises, which was transferred to and analyzed in the cloud. Using the results of the analysis, clinicians were able to monitor the progress of their patients remotely.	Compared to in-person (and phone-based) rehabilitation, VRehab resulted in a steady increase in Mobility Index and at least one stage improvement in Brunnstrom Stage.
Ghorbel et al.^58^	To examine the impact of color-based 3D skeletal feedback to guide patients in completing rehabilitation exercises.	In a desktop application, color-based 3D skeletal feedback was superimposed on the videos of patients to guide them in completing exercises. Additionally, the movements of the patients were automatically analyzed and reported to clinicians.	The visual feedback improved the posture of the patients and enhanced the motion in the case of simple exercises. The VRehab platform was reliable, simple to use, and positively impacted patients' psychology measures. Clinicians and patients both found the measurement and feedback to be accurate, reliable, and safe.
Qiu et al.^57^	To evaluate the feasibility of VRehab platform to prepare for a future efficacy study.	Patients controlled the rehabilitation game with the Leap Motion controller on their hand, and the difficulty of the game was determined adaptively according to the movements of patients. The movement data was transferred to the cloud, where clinicians could view it.	Patients were able to use the VRehab platform resulting in improvements in Upper Extremity Fugl-Meyer and hand kinematics.
Sobrino et al.^56^	To evaluate the perceived usefulness and ease of use of VRehab platform.	The movements of patients were analyzed, and accordingly, on-screen textual and visual feedback was provided to patients regarding the quality of their exercises. Movement analysis results were also reported to clinicians.	The collected questionnaire data regarding the perceived usefulness and ease of use of the platform indicated a positive view of patients.
Triantafyllidis et al.^66^	To evaluate the feasibility of VRehab platform.	In response to the real-time sensor data collected, a virtual coach was animated to provide patients with safe and personalized exercise feedback within their beneficial heart rate zones.	With the assistance of the virtual coach, patients were able to exercise within or above their beneficial heart rate zones for the majority of the exercise duration.
Yu et al.^65^	To develop a remote quantitative Fugl-Meyer assessment framework,	The collected data from a wearable sensor network was used to automatically measure the Fugl-Meyer score.	The proposed quantitative models could precisely predict the Fugl-Meyer assessment based on wearable sensor data.
Zhang et al.^64^	To develop and evaluate a wearable exoskeleton rehabilitation robot for clinic and home-based rehabilitation.	A wearable exoskeleton rehabilitation robot, along with a 3D animation, was used to perform task-based repetitive therapy.	Significant improvements in both the Wolf Motor Function Test and Fugl–Meyer Assessment scores were reported for some patients in both clinical and home settings.

**Table 2:** Characteristics of virtual rehabilitation programs in the included studies. An “NA” indicates that the corresponding item was not addressed or discussed in the paper.

Authors	Rehabilitation type	Program duration (weeks)	Number of sessions (per week)	Session duration (minutes)	Metric to evaluate the delivery of rehabilitation	Metric to compare to in-person rehabilitation	Medium of Delivery
Gupta and Kohli^63^	Physiotherapy	12	2-3	45-60	Hospital readmission rate	NA	Smartphone application
Kohli and Gupta^62^	Physiotherapy	12	2-3	45-60	NA	NA	Smartphone application
Fabio et al.^61^	Motor and Cognitive	10	3	60	Improvement in cognitive and motor performance	NA	Web application and desktop application
Bo et al.^25^	Stroke	6	NA	NA	Improvement in the Wolf Motor Function Test, the Fugl–Meyer Assessment, and the Mattis Dementia Rating Scale	NA	Smartphone application
Bouteraa et al.^26^	Wrist	1.5	10 overall	NA	NA	NA	Desktop application and robot
Tsvyakh et al.^60^	Orthopedic	3	NA	NA	The monitoring of exercise time, local temperature, the biomechanics of active movements of the injured limb	Patient satisfaction and orthopedic surgeon visit time	Smartphone application and wearable device
Fang et al.^59^	Stroke	54.5	442 overall	NA	Brunnstrom Stage and Mobility Index	Brunnstrom Stage and Mobility Index	Web application
Ghorbel et al.^58^	Stroke	3	3	NA	Time Variable Replacement-based average distance, average postural angles	NA	Desktop application
Qiu et al.^57^	Upper extremity stroke	12	7	15	Upper Extremity Fugl-Meyer and hand kinematics	NA	Desktop application
Sobrino et al.^56^	Physical	NA	NA	NA	NA	NA	Web application
Triantafyllidis et al.^66^	Cardiac	4	43	30	The percentage of exercises that can be completed within or above patients’ beneficial HR zones	NA	Desktop application
Yu et al.^65^	Stroke	13	1	NA	NA	NA	Desktop application
Zhang et al.^64^	Stroke	4	7	45	Improvement in Wolf Motor Function Test and Fugl–Meyer Assessment	Improvement in Wolf Motor Function Test and Fugl–Meyer Assessment	Desktop application and wearable robot

**Table 3:** Characteristics of artificial intelligence in the included studies. An “NA” indicates that the corresponding item was not addressed or discussed in the paper.

Authors	Sensors for data collection	Timing of data analysis	AI algorithm for data analysis	The outcome of AI algorithms
Gupta and Kohli^63^	RGB camera	Retrospective	AI Algorithms in the TheraNow application for physical therapy	NA
Kohli and Gupta^62^	RGB camera	Retrospective	AI Algorithms in the TheraNow application for physical therapy	NA
Fabio et al.^61^	RGB camera	Concurrent	Eye gaze and skeleton extraction	NA
Bo et al.^25^	Built-in smartphone sensors for motion analysis	Retrospective	Multiple linear regression and random forest	predicting the percentage difference of Wolf motor function test
Bouteraa et al.^26^	Current sensor, electromyography sensor, RGB camera, and Kinect camera	Concurrent	Fuzzy inference	Degree of pain
Tsvyakh et al.^60^	Axis, temperature, and volume sensors	Concurrent	NA	NA
Fang et al.^59^	Inertial measurement unit based body sensor network	Concurrent	K-nearest-neighbor classifier and fuzzy inference system	Brunnstrom Stage and Mobility Index
Ghorbel et al.^58^	Kinect depth camera	Concurrent	Template matching	Correctness of rehabilitation exercises
Qiu et al.^57^	Leap motion controller	Concurrent	Key press rating tracking	Determining the difficulty of exercise
Sobrino et al.^56^	Kinect depth camera	Concurrent	Dynamic time warping	Distance between patient’s exercise and therapist’s exercise
Triantafyllidis et al.^66^	Kinect depth camera, wristband for heart rate measurement, and blood pressure monitor	Concurrent	Rule-based system for guiding patients in doing exercises	Providing guidance for patients in doing exercises
Yu et al.^65^	Accelerometer and flex sensors	Retrospective	Extreme learning machine	Fugl-Meyer assessment (regression)
Zhang et al.^64^	Sensing through wearable robots	Concurrent	Motion detection	NA

#### Characteristics of studies

The included studies (*n* = 13) were published between 2011 and 2022 with the majority of the studies, 10 (76.9%), having been published between 2020-2022^25,27,56–63^ which shows the shift in the use of VRehab and AI solutions across many populations to support rehabilitation of people living in the community. Among the included studies, 5 (38.5%) were conducted in the United States^25,57,62–64^, 2 (15.4%) in China^59,65^, 2 (15.4%) in Spain^56,58^, one (7.7%) in Greece^66^, one (7.7%) in Italy^61^, one (7.7%) in Tunisia^27^, and one (7.7%) in Ukraine^60^. Of the included studies, 12 (92.3%) were journal articles and 1 (7.7%) was a peer-reviewed conference publication. These studies were published in multidisciplinary digital health or biomedical, or single-disciplinary engineering journals or conferences.

#### Characteristics of participants

The inclusion criteria specified the use of AI-driven VRehab platforms for patients or mixed (patients and healthy) populations; however, none of the included studies incorporated healthy individuals (along with patients). Six (46.2%) of the studies were stroke rehabilitation for acute and chronic stroke patients^25,57–59,64,65^, 5 (38.5%) of the studies were physical therapy rehabilitation for post-hip and knee-replacement surgeries^62,63^, wrist fracture^27^, polytrauma lower extremities^60^, and musculoskeletal injuries patients^56^, one (7.7%) of the studies were motor and cognitive rehabilitation for Rett syndrome patients^61^, and one (7.7%) focused on exercise-based cardiac rehabilitation for cardiovascular disease patients^66^. The age of participants in most of the studies was around 60 years old, involving late middle age and late adulthood, with only one study on Rett syndrome patients^61^ and one on musculoskeletal injuries patients^56^ involving early adulthood. Except for two single-sex studies^27,61^, all other studies recruited both sexes, with *n* = 139 (50.4%) females and *n* = 137 (49.6%) males across all the included studies.

#### Characteristics of rehabilitation programs

In 8 (61.5%) of the 13 included studies^25,27,56–58,62,63,65^, the same setting of home-based VRehab program was provided to all study participants. However, in 5 (38.5%) of the included studies^59–61,64,66^, participants were divided into two groups and received two different rehabilitation programs. Triantafyllidis et al.^66^ evaluated their VRehab platform in an in-person simulation setting with 10 (76.9%) patients and in patients’ homes in a real-world setting with 3 (23.1%) patients. Tsvyakh et al.,^60^ Fang et al.^59^, and Zhang et al.^64^ randomly recruited patients to participate in home-based or in-person rehabilitation. Fabio et al.^61^ used VRehab with no AI, including regular video calls between patients and clinicians, for 10 (50.0%) and AI-driven VRehab for the other 10 (50.0%) patients.

### Synthesis of results

#### Research Question 1: How was AI applied in the delivery of home-based VRehab programs to patients living in the community?

This subsection describes various sensing modalities used for data collection and providing input to AI algorithms, characteristics of AI algorithms, the outcome of AI algorithms, and the usage of the outcome of AI algorithms in VRehab programs.

**Sensors and input data to AI algorithms:** In the majority of the included studies, different sensors were used to collect data on patients’ movement during rehabilitation exercises. This data collection was in line with the study’s goal of providing guidance and feedback to patients during their exercise routines.^25,27,56–66^. To monitor other health indicators of patients during exercises, two studies were also equipped with physiological sensors^60,66^ along with sensors for capturing body movements. Due to the availability and ubiquity of regular RGB cameras available on smart devices available at home (PC, laptop, smartphone, and tablet), it is the most common sensing device for data acquisition (*n* = 4)^27,61–63^. RGB cameras may suffer from capturing improper body movement data in the wild (at home) because of issues with their sensitivity to light, brightness, camera angle, and privacy. As an alternative, *n* = 4 studies used the Kinect depth camera^27,56,58,66^, which can overcome some of the challenges imposed by RGB cameras; however it is an external piece of hardware with an additional cost. Other types of sensors to capture body movement data included smartphones’ built-in Inertial Measurement Units (IMU)s (*n* = 1)^25^ or standalone sensors such as accelerometers (*n* = 1)^65^, flex (*n* = 1)^65^, and leap motion sensors (*n* = 1)^57^.

The sensors for collecting physiological data included wristband sensors, such as heart rate or standalone blood pressure monitors^66^. Some studies also utilized sophisticated sensors to collect data from patients at home, such as wearable robots^64^ or IMU body sensor networks^59^.

**Characteristics of AI algorithms:** The data collected from patients at home through the aforementioned sensors served as input for AI algorithms, enabling the derivation of valuable inferences about patients. Sensors collecting data and AI algorithms making inferences using the collected data acted as a proxy for clinicians who were not physically present in VRehab programs.

Bo et al.^25^ developed a feature-based machine-learning approach for predicting the percentage difference of Wolf motor function test in stroke survivors at different timestamps of their rehabilitation program. Various features in four categories of movement phenotyping, compliance, clinical, and demographic were collected from patients in VRehab. Movement phenotyping features were collected by smartphone built-in sensors, containing information regarding the number of days patients completed their prescribed exercise, the number of repetitions of exercises in a day, the duration of exercise sessions, and many others. Compliance features were calculated based on the number of days and number of sessions for a specific time duration. Clinical features were the clinical assessment information, such as the Fugl-Meyer assessment score and the total months the patients had a stroke. Demographic features were age and sex. Various combinations of the above features were examined to build predictive machine-learning models for the percentage difference in the Wolf motor function test in different periods of the VRehab program. Combining all the features in the above four categories was found to significantly improve the performance of the predictive models. The machine-learning models were multiple linear regression and random forest, with the latter resulting in lower root mean square error.

Bouteraa et al.^27^ developed a feature-based decision support system based on cascading fuzzy logic algorithms to measure the degree of pain in wrist fracture patients in exercise sessions of VRehab and control an exercise rehabilitation robot accordingly. In addition to visual movement data collected through RGB and depth cameras, various time-domain and frequency-domain features were extracted from current and electromyography sensors on the robot. The extracted features were input to cascades of fuzzy logic algorithms to output the degree of pain.

Fang et al.^59^ developed a feature-based approach for Brunnstrom Stage and Mobility Index classification. Acceleration signals containing movement information of patients while doing rehabilitation exercises were collected using an IMU-based body sensor network. The signals were segmented into individual exercise repetitions through peak detection. Dimensionality reduction was applied to the signals using principal component analysis and input to an adaptive neuro-fuzzy inference system for Brunnstrom stage classification.

Ghorbel et al.^58^ evaluated the quality of rehabilitation exercises completed by patients by comparing and calculating the distance between Kinect body joint data of patients’ exercises with Kinect body joint data of reference correct exercises. Thresholding the calculated distance resulted in a decision regarding the correctness of patients’ exercises. According to the decision, on-screen visual feedback was provided to patients.

Qiu et al.^57^ used a cloud-based AI algorithm for measuring and tracking key press rate working on a leap motion controller. The measured rate was used to adaptively determine the difficulty of hand exercises for stroke patients.

Sobrino et al.^56^ evaluated the quality of patients’ rehabilitation exercises by comparing and calculating their distance with therapist’s exercises as references. The distance was a measure of exercise quality and was used to provide real-time textual feedback to patients.

Triantafyllidis et al.^66^ developed a feature-based approach for exercise quality assessment. Different features were collected from various sensors, including a Kinect depth camera, wristband for heart rate measurement, and blood pressure monitor, and classified by a rule-based algorithm to output exercise quality. Accordingly, visual feedback in the form of an animated avatar was provided to patients.

Yu et al.^65^ developed a feature-based method for the Fugl-Meyer assessment as a regression problem. Various features, including amplitude, mean value of sensor data, root mean square value, root mean square value of the derivative, and approximate entropy were extracted from accelerometer and flex sensor signals. These features were input to an extreme learning machine regression model to perform the Fugl-Meyer assessment.

Some of the reviewed studies did not mention the details of their AI algorithms, such as eye gaze and body joints skeleton extraction from video^61^, the algorithm for tracking key press rating^57^, or motion detection^64^. Two studies identified the name of an AI-powered smartphone application with no details of the AI algorithms used in the application^62,63^.

**AI algorithms’ outcomes and their usage:** The outcomes of AI algorithms were found to be primarily related to patients’ movements and exercises, including the correctness of rehabilitation exercises^58^, the distance between the exercise performed by the patient and the exercise performed by the clinician^56^, the percentage difference of Wolf motor function test^25^, Brunnstrom Stage^59^, Mobility Index^59^, Fugl-Meyer assessment^25,57,64,65^, and the degree of pain during exercises^27^. Only three studies followed reporting standards and explained the details and the training and evaluation phases and performance metrics of their AI algorithms, including root mean square error^25,65^, distance^58^, coefficient of determination^65^, and training time^65^. Two studies that used commercial products did not provide details of the AI algorithms in their product^62,63^.

The outcomes of AI algorithms were used in a variety of ways, primarily for prescribing individualized rehabilitation exercises to patients^27,62–64^, providing visualizations and feedback to patients in completing their exercises^27,56–58,62,63,66^, and providing reports to clinicians about the progress of patients in their rehabilitation program^25,27,56–65^. Other uses of the outcomes of AI algorithms included attentiveness assessment of patients based on face and eye features^61^ and pain assessment^27^. The mediums to deliver feedback and visualizations to patients included desktop application^27,57,58,61,64–66^, smartphone application^25,60,62,63^, web application^56,59,61^, and wearable robot^27,64^.

#### Research Question 2: How effective was the application of AI in the delivery of home-based VRehab programs for patients living in the community?

This subsection describes how the effectiveness of AI-driven VRehab platforms in the delivery of rehabilitation to patients’ homes was evaluated and how effective they were found to be.

**Metrics for effectiveness:** A wide variety of metrics were used to evaluate the effectiveness of AI-driven VRehab platforms, including hospital readmission rate^63^, patient satisfaction^60,62^, perceived usefulness^56^, perceived ease-of-use^56,59^, reduction in clinician consultation time^60^, and various disease-specific assessment metrics, e.g., stroke-specific assessments, including Wolf Motor Function Test^25,64^, and the Fugl-Meyer Assessment^25,57,64,65^.

**Evaluation of effectiveness:** As described above, none of the reviewed studies included healthy populations in their cohort along with rehabilitation patients. The patient population used the same or different settings of rehabilitation programs. In the studies with the same rehabilitation setting, there was no comparison between virtual/in-home and in-person/in-hospital rehabilitation. The majority of the included studies provided all patients with the same rehabilitation program, AI-driven VRehab at home^25,26,56–58,62,63,65^. Few of these studies investigated the comparison of their outcomes with those reported in the literature^62,63^. As an example, the Net Promoter Score (NPS) is a metric for patient satisfaction and recommendation to others^62^; it was much higher than the average NPS score for the healthcare industry. Therefore, it was concluded that the VRehab platform was pleasing to patients^62^. The hospital readmission rate in 30 days of total hip and knee replacement post-surgical follow-up was compared with the reported hospital readmission rate in the previous literature and based on its lower values, the effectiveness of the AI-driven VRehab platform was concluded^63^. Qiu et al.^57^ reported 100% rehabilitation program completion and improvements in upper extremity Fugl-Meyer assessment for all the patients in their study.

In other studies with the same VRehab settings for all patients^25,27,65^, the VRehab platform’s effectiveness in delivering rehabilitation was not reported. However, the performance of the AI algorithms in predicting an outcome variable was reported. Ghorbel et al.^58^ investigated the correctness of exercises of the same patients with and without visual feedback on the computer and reported lower distances between correct exercises and patients’ exercises when visual feedback was provided to patients. Qualitative measures were also reported from patients’ perspectives regarding the visual feedback provided to patients which included: Posture correction as a strength of the system, usefulness of the feedback, relevance of measurement performed by the VRehab platform, reliability and simplicity of the system, safety of the platform, interestingness and not being tiring of the exercise^58^.

Fabio et al.^61^ reported improvements in neuropsychological assessments for Rett syndrome patients when using AI-driven VRehab compared to non-AI-driven VRehab. Triantafyllidis et al.^66^ evaluated their VRehab platform in an in-person simulation setting and patients’ homes in a real-world setting. The patients were able to perform most of the cardiac rehabilitation exercises within or above their beneficial heart rate zones. Tsvyakh et al.^60^, Fang et al.^59^, and Zhang et al.^64^ randomly recruited patients to participate in home-based or in-person rehabilitation. Tsvyakh et al.^60^ reported much less clinician-patient visit time and much higher patient satisfaction in VRehab compared to in-person rehabilitation. Ghorbel et al.^58^ reported a steady increase in the Mobility Index and at least one stage improvement in Brunnstrom Stage VRehab compared to in-person (and phone-based) rehabilitation. Zhang et al.^64^ reported significant improvements in the Wolf Motor Function Test and Fugl-Meyer Assessment scores for some patients in both in-person and virtual in-home settings.

## Discussion

In this scoping review, thirteen studies were identified that reported the application of AI-driven VRehab platforms in the delivery of rehabilitation services to patients in their homes. The studies made use of a variety of sensors to collect data about patients in different modalities. The collected data were used by AI algorithms to provide guidance and feedback to patients or report patients’ performance to clinicians. A variety of effectiveness evaluation metrics revealed that AI-driven home-based VRehab was effective in improving patients’ health outcomes compared to non-AI-driven home-based VRehab (n = 1) and in-person rehabilitation (n = 4). There was a clear indication that patients were satisfied using these platforms in terms of high levels of reported satisfaction and corresponding improvements in disease-specific assessment metrics. Our fidings also indicated a paucity of research focused specifically on the evaluation of the effectiveness of integrating AI with VRehab for patients in their homes.

### Challenges, limitations, and recommendations

#### Reporting VRehab characteristics

Most of the reviewed studies reported baseline demographic information of patients^67,68^, such as age, sex, medical condition or diagnosis, comorbidity, marital status, employment status, income, socioeconomic status, and health insurance coverage. However, barriers influencing patient adherence to both in-person and VRehab programs were not reported including^2,69^: transportation issues, family obligations, lack of motivation and energy, and finding rehabilitation exercises tiring and painful. The fact that these variables are associated with adherence to rehabilitation programs necessitates the incorporation of these variables into studies that examine the effectiveness of AI-driven platforms for rehabilitation delivery^2^. More importantly, home-based- and virtual-specific information or barriers to adherence to and completion of VRehab programs need to be collected and reported. These potential barriers include sensor and smart device installation and maintenance costs, internet connection stability^70^, minimum system requirements of computers and smartphones for VRehab applications, type of residence (e.g., house, townhouse, apartment, community housing, or basement), digital literacy or computer skills of patients^57^, and hearing or vision impairments. Among the reviewed home-based VRehab studies, only Qui et al.^57^ reported some of the barriers noted above, including residence type and computer skills.

#### Infrastructure barriers

A major roadblock to the adoption of VRehab is that all patients may not have access to the digital devices and internet connectivity required to participate in these programs^71,72^. This is particularly relevant in low-income communities, rural areas, and among certain patient populations such as ethnocultural minorities. Policymakers and developers of VRehab programs need to ensure inclusivity in their strategic planning to facilitate VRehab programs that are accessible and improve health outreach among diverse patient populations. Policymakers are encouraged to implement equitable digital health solutions, such as subsidizing required digital devices and internet connectivity for those who are unable to afford these services. This could involve partnerships with local governments, non-profits, and other organizations to provide low-cost or free devices and connectivity to patients in need. Engineers can develop less sophisticated products at lower costs. For example, instead of using depth cameras for the extraction of body joints of patients while exercising at home^27,56,58,66^, a regular built-in RGB camera in laptops and smartphones, can be used along with advanced deep-learning methods for body joint extraction from RGB video^37^.

#### Co-design

A significant aspect not considered in the reviewed studies was co-design or patient-centric participatory design. Co-design involves the inclusion of patient partners and clinicians from the outset in the design of various modules of VRehab platforms, including the user interfaces and functionality of applications, wearables, and other devices required to deliver VRehab at home^73,74^. In a general co-design framework, there is ongoing feedback and iterative discussion between patients, clinicians, and researchers with the aim of improving the development, design, and usability of VRehab platforms and incorporating patients’ views during the process. By utilizing a co-design framework, the VRehab platform will be more usable, effective, motivational, engaging, and customized to meet the specific needs of clinicians and patients^75^. Furthermore, a co-design approach is essential since VRehab platforms are intended for use by patients at home without the presence or supervision of clinicians. It is noteworthy that co-design should take place during initial development and prior to the deployment of VRehab solutions. Due to the aforementioned challenges, including infrastructure and digital literacy limitations of patients in independently engaging with VRehab platforms at home, co-design sessions are predominantly conducted on-site or within a controlled laboratory setting. This arrangement enables researchers and developers to closely interact with patients and stakeholders, facilitating active participation, feedback, and iterative refinement of the VRehab platform^76,77^.

#### Usability, acceptability, and safety

There is no validated scale available to measure the usability, acceptability, and safety of VRehab platforms. Commonly used scales to measure people’s perceived usability of digital systems, such as the System Usability Scale^78,79^ are not tailored to VRehab platforms or patient populations and were designed for general use. Consequently, researchers have developed a variety of evaluation scales specific to their platforms^56,57,60,62,63^. For instance, Sobrino et al.^56^ developed their own questionnaires to evaluate the “perceived usefulness" and “perceived ease-of-use" of their VRehab platform. This incoherence warrants more research to develop validated usability scales tailored specifically for VRehab platforms. Moreover, the safety of VRehab platforms is a critical aspect that requires rigorous evaluation. Presently, there are limited studies assessing the potential risks and safety protocols specific to VRehab settings^80,81^. This gap indicates a need for comprehensive safety guidelines and standardization in VRehab platforms to ensure patient well-being and trust in these emerging technologies. Furthermore, exploring user feedback and incident reports can provide valuable insights into the safety challenges and areas for improvement in VRehab platforms.

#### Privacy and personalization

Preserving patients’ privacy and the personalization of AI models for individual patients^82,83^ are critically important aspects that were not addressed or discussed in the included studies. In contrast to traditional on-site rehabilitation, VRehab collects data from patients at their homes. The collected data must be transferred to a central location/cloud via the Internet in order to be used for the development of AI models. However, sharing patient data over the Internet raises concerns about privacy and potential information leaks^84^. In the case that patients’ collected data is transferred to a central location/cloud or collected in a centralized manner (e.g., data collection from patients in hospitals) and used for AI model development, trained AI models should be personalized for individual patients at home using their personal data^82^. Privacy-preserving machine learning techniques such as federated learning^83^ and split learning^84,85^ can be employed. These techniques facilitate AI model training and AI model personalization in a decentralized manner without the need to share raw data from patients over the Internet. However, it is important to note that implementing these privacy-preserving techniques requires computers at patients’ homes with sufficient computational power for local model training^86^.

#### Can VRehab replace clinicians?

AI and VRehab have been posited as potential replacements for clinicians in certain healthcare settings, which has sparked debate in the field^5,87^. Some studies suggest that AI-driven VRehab platforms can automate repetitive tasks and identify patterns, reducing physical contact between clinicians and patients^8,10,18–20,44,45,87,88^. However, critics argue that AI and VRehab technologies cannot replace the expertise of trained clinicians in complex assessments and decision-making, as well as providing emotional support to patients^89^. Furthermore, these technologies may introduce bias and errors that could threaten patient safety^8,10,18–20,44,45,88^. Therefore, it is suggested that AI and VRehab should supplement rather than replace clinicians, in order to enhance the care they provide. AI-driven VRehab platforms have the potential to address the shortage of rehabilitation staff and improve operational efficiencies, thus increasing access to rehabilitation care for a larger patient demographic^3,5,7,90^. However, the reviewed studies lack an analysis of how much VRehab platforms can reduce clinician intervention or on-site patient visits^5^. In only one study^60^, clinicians were reported to spend less time visiting patients in VRehab as compared to in-person rehabilitation.

The majority of the reviewed studies used traditional machine-learning approaches to make inferences regarding patients’ health outcomes in VRehab programs. Recent advances in computer vision and signal processing have demonstrated that deep learning can outperform traditional machine learning techniques. Therefore, it is recommended that future studies examine the use of deep learning algorithms to improve existing state-of-the-art methods. For deep learning algorithms to build meaningful predictive models, large amounts of data are usually required^91^, and in many cases, a sufficient population might not be readily available. In addition, deep learning algorithms require expensive hardware to run, and the models may be uploaded to the cloud, which would incur additional costs and privacy concerns.

This review benefits from the use of a systematic, reproducible process guided by an established scoping review framework^51^. In order to ensure a thorough and comprehensive examination of relevant literature, the search strategy used in this review was developed in consultation with a Library Sciences Expert. In addition, this review spans studies from the inception of this technology to the present, ensuring that all pertinent literature was included. Although a thorough and comprehensive search was completed, additional articles and resources may have been missed due to the exclusion of non-English language articles. Another limitation of this review is the heterogeneity in outcome measurement tools, outcome assessment times, patient populations, and randomization methods among the studies examined, which precluded meta-analysis.

## Conclusion

Personalized and ambulatory rehabilitation services can be delivered to patients at home by integrating AI into VRehab platforms. AI algorithms are able to make individualized and real-time inferences about patients’ rehabilitation progress based on data collected from various sensors. Since improving functional and mobility outcomes is the central focus of rehabilitation programs for different patient populations, the majority of the studies reviewed targeted facilitating prescribed exercise completion at home in the absence of clinicians. In almost all of the reviewed studies that assigned participants to different rehabilitation settings, AI-driven home-based VRehab was found to be more effective than in-person rehabilitation and non-AI-driven home-based VRehab. The feasibility, safety, and privacy implications of AI-driven VRehab platforms still warrant further investigation. Researchers in this field must also be cognizant of the potential ethical and legal implications associated with the application of AI in VRehab. In order to address the current limitations and fully realize the potential of AI-driven VRehab, it is crucial that interdisciplinary collaboration is fostered.

### Reporting summary

Further information on research design is available in the Nature Research Reporting Summary linked to this article.

### Supplementary information

Supplementary Table 1

Reporting Summary

## Data Availability

All data generated or analyzed during this study are included in this published article.
