# Supplementary material for: Artificial intelligence-driven virtual rehabilitation for people living in the community: A scoping review
Source: NPJ Digit Med. 2024 Feb 3;7:25. doi: 10.1038/s41746-024-00998-w (PMC10838287; doi:10.1038/s41746-024-00998-w)
Supplement: Supplementary file 1 — Supplementary Table 1 [file 41746_2024_998_MOESM1_ESM.pdf]

**Supplementary Table 1.** Database Search String.	
Database	Search string
Medline and Embase	(rehabilitation/ OR cardiac rehabilitation/ OR stroke rehabilitation/ OR occupational therapy/ OR physical therapy/ OR exp exercise therapy/ OR telerehabilitation/ OR (rehab* OR telerehab* OR tele-rehab* OR virtual rehab* OR e-rehabl).tw,kw. OR (therap* adj3 (physical OR occupational OR exercis* OR virtual)).tw,kw. OR (physiotherap* OR kinesiotherapl.tw,kw.) AND (exp remote consultation/ OR home care services/ OR ((rehab* OR therap*) adj4 (home* OR virtual OR "in-home" OR "at-home")).tw,kw. OR (telerehab* OR tele* rehab* OR tele-rehab* OR web-based OR internet* OR e-rehab* OR tele-consult* OR teleconsult* OR tele* consult*).tw,lcw. OR (remote adj3 (rehab* OR physiotherapy* OR kinesiotherap* OR physical therap* OR monitor*)).tw, kw. OR (virtual adj3 (physiotherap* OR kinesiotherap* OR physical therap* OR pt OR environment* OR monitort)).tw,kw.) AND (artificial intelligence/ OR exp machine learning/ OR algorithms/ OR Pattern Recognition, Automated/ OR Signal Processing, Computer-Assisted/ OR (affective adj3 (state? OR computl).tw, kw. OR (artificial intelligence* OR ambient intelligence* OR machine intelligence* OR machine learning* OR deep learning* OR algorithm* OR sensing system* OR wearable monitor* OR physiology sensor* OR computer vision* OR artificial neural network* OR motion data* OR vector machine* OR signal processing*).tw,kw. OR (recognition adj4 (locomot* OR gesture OR automatic OR pain* OR tired* OR engage* OR pattern* OR active OR exercis* OR pain* OR tired* OR technolog*)).tw,kw. OR ((sensor* OR device* OR monitor*) adj3 (wearable* OR Kinect OR video* OR cameral).tw,kw. OR (pattern* adj3 (motion OR actIon)).tw,lcw. OR (technology* adj3 (solution* OR physical* actin* OR feedback*)).tw,kw. OR (data adj3 (motion OR stride)).tw,kw. OR (motion* adj3 (capture OR tracking)).tw,kw.)
Web of Science	((rehab* OR telerehab* OR tele-rehab* OR "virtual rehab*" OR e-rehab*) OR (therap* NEAR/3 (physical OR occupational OR exercise* OR virtual)) OR (physiotherap* OR kinesiotherap*)) AND (((rehab* OR therap*) NEAR/4 (home* OR virtual* OR "in-home" OR "at-home")) OR (telerehab* OR "tele* rehab*" OR tele-rehab* OR web-based OR internet* OR e-rehab* OR tele-consult* OR teleconsult OR "tele* consult*") OR (remote NEAR/3 (rehab* OR physiotherap* OR kinesiotherap* OR "physical therap*" OR monitor*)) OR (virtual NEAR/3 (physiotherap* OR kinesiotherap* OR "physical therap*" OR PT OR environment* OR monitor*))) AND ((affective NEAR/3 (state? OR compue*)) OR ("artificial intelligence*" OR "ambient intelligence*" OR "machine intelligence*" OR "machine learning*" OR "deep learning*" OR algorithm* OR "sensing system*" OR "wearable monitor*" OR "physiology sensor*" OR "computer vision*" OR "artificial neural network*" OR "motion data*" OR "vector machine*" OR "signal processing") OR (recognition NEAR/4 (locomot* OR gesture OR automatic OR pain* OR tired* OR engage* OR activit* OR assessment* OR technolog* OR gait OR motion OR pattern) OR TS=(detect* NEAR/4 (emotion' OR "human pose" OR tracking OR locomot* OR motion OR gait OR pattern* OR activie* OR exercis* OR pain* OR tired* OR technolog*) OR ((sensor* OR device* OR monitor*) NEAR/3 (wearable* OR Kinect OR video* OR camera*)) OR (pattern* NEAR/3 (motion OR action)) OR (technology* NEAR/3 (solution* OR "physical* activ*" OR feedback*)) OR (data NEAR/3 (motion OR stride)) OR (motion* NEAR/3 (capture OR tracking)))
IEEE Xplore	("All Metadata":rehabilitation) AND ("All Metadata":virtual) AND ("All Metadata":"artificial intelligence")
Google Scholar	virtual rehabilitation artificial intelligence - virtual rehabilitation machine learning - virtual rehabilitation deep learning - telerehabilitation artificial intelligence - telerehabilitation machine learning - telerehabilitation deep learning - home rehabilitation artificial intelligence - home rehabilitation machine learning - home rehabilitation deep learning
